# Suspected Congenital Absence of the Vermiform Appendix in a 13-Year-Old Girl: A Case Report

**DOI:** 10.7759/cureus.113918

**Published:** 2026-08-04

**Authors:** Farooq Malik, Misbah Arif, Azka Irshad, Hafeez Nassar, Areena Arshad, Muhmmad Adeel

**Affiliations:** 1 General Surgery, Medicsi Hospital, Rawalpindi, PAK; 2 General Surgery, Quaid-e-Azam International Hospital, Rawalpindi, PAK; 3 General Surgery, Medicsi Hospital Islamabad, Islamabad, PAK; 4 Surgery, Loralai Medical College, Loralai, PAK; 5 Surgery, Poonch Medical College, Rawalakot, PAK; 6 Surgery, Al-Nafees Medical College, Islamabad, PAK

**Keywords:** acute appendicitis, appendiceal agenesis, congenital absence of appendix, congenital hypoplasia, pediatric surgery, vermiform appendix

## Abstract

A congenital absence of the vermiform appendix (appendiceal agenesis) is a rare anatomical anomaly that is mostly recognised incidentally upon exploratory surgery due to suspected appendicitis. Preoperative diagnosis is difficult since symptoms and imaging studies typically resemble other causes of right lower quadrant pain. In this report, we describe an adolescent girl aged 13 who presented with right lower quadrant abdominal pain; this led to a workup to rule out acute appendicitis. Imaging studies were performed to evaluate the cause of her pain. An ultrasound examination of the abdomen demonstrated an enlarged left ovary (bulky), and a contrast-enhanced computed tomography (CT) scan of the abdomen/pelvis confirmed that there was a left ovarian cyst (3-4 cm in diameter) along with a partially calcified lesion involving the left adnexa and that the ileocaecal region appeared to be normal. Conservative treatment was not completely effective in eliminating the symptoms. Thus, surgical exploration was decided. During the operation, a small amount of reactive liquid was found in the ileocaecal area. A comprehensive and careful search of the ileocecum, followed by tracing of the taenia coli, did not reveal the presence of a vermiform appendix, and only a small 1 cm nodular structure was found at the end of the taenia coli. Congenital hypoplasia of the appendix was strongly suspected based on intraoperative findings, and hence, abdominal closure was performed without further complications. The postoperative course was uneventful. The case demonstrates how difficult it can be to diagnose appendicitis when clinical findings and imaging studies present ambiguity in diagnosis.

## Introduction

Congenital agenesis of the vermiform appendix is one of the rarest congenital abnormalities, occurring in about 1 per 100,000 explorative laparotomies conducted due to suspicion of acute appendicitis [1]. The vermiform appendix arises as a blind-ending diverticulum derived from the posteromedial wall of the cecum during development [2].

Patients with congenital absence of the appendix manifest with clinical symptoms similar to those associated with acute appendicitis, making the presurgical diagnosis of the condition highly challenging [3]. Although both ultrasonography and computed tomography (CT) play an essential role in identifying inflammation in suspected appendicitis, these tests cannot be used for diagnosing the absence of the appendix prior to surgery [4]. Moreover, the presence of numerous diseases may simulate appendicitis, such as Meckel's diverticulitis, mesenteric adenitis, Crohn's disease, ovarian torsion, rupture of ovarian cysts, pelvic inflammatory disease, intussusception, and pathology of the genitourinary tract [5]. As a result, congenital absence of the appendix can be confirmed only during surgery after excluding other surgical causes of pain in the right iliac fossa.

This report describes a 13-year-old female who presented with clinical features suggestive of acute appendicitis but was found intraoperatively to have congenital absence of the vermiform appendix. By highlighting the diagnostic uncertainty, the systematic intraoperative evaluation required to exclude alternative pathologies, and the implications for surgical decision-making, this case contributes to the limited literature on appendiceal agenesis and emphasises the importance of considering this uncommon anomaly when the appendix cannot be identified during surgery.

## Case presentation

A 13-year-old female was admitted to the emergency department with lower abdominal pain radiating to the right iliac fossa, accompanied by nausea but without vomiting or fever. Furthermore, she was complaining of an increased frequency of micturition without dysuria. She did not have any significant medical or surgical past history.

On examination, she had lower abdominal tenderness. Investigations revealed total leucocytosis of 10.7 × 10³/µL and a haemoglobin level of 11.6 g/dL. Urinalysis was normal (Table 1). Abdominal ultrasonography revealed normal findings, with a simple cyst of the left adnexa measuring 34 × 36 mm (28 mL).

**Table 1:** Laboratory investigations at presentation, including patient values, reference ranges, and clinical interpretation.

Parameter	Patient Value	Reference Range	Interpretation
Haemoglobin	11.6 g/dL	12.0–16.0 g/dL	Mildly decreased
Total leukocyte count	10.7 × 10³/µL	4.0–11.0 × 10³/µL	Within normal range (upper limit)
Neutrophils	55%	40%–70%	Within normal range
Urine routine examination	Normal	Normal	No abnormality detected

**Figure 1:**
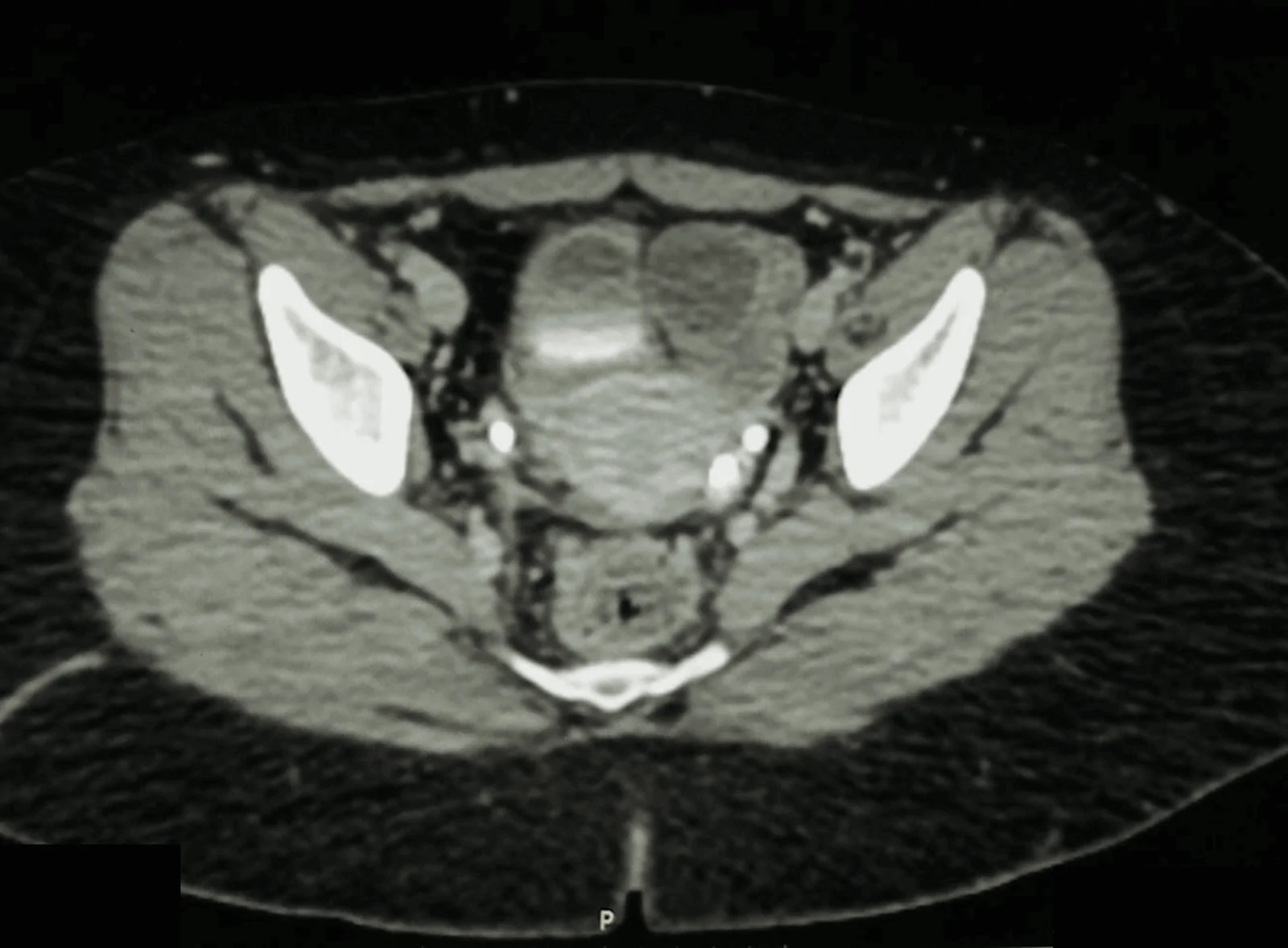
Axial contrast-enhanced computed tomography (CT) image of the pelvis showing the left adnexal cystic lesion (arrow) with adjacent ill-defined calcific foci in the left adnexal region. No significant abnormalities are identified within the visualised bowel loops or ileocaecal region.

She was treated with intravenous ampicillin and metronidazole along with analgesics and discharged from the hospital. Since her symptoms were recurring and she did not improve, a contrast-enhanced CT scan of the abdomen and pelvis was done on Day 2. On the CT scan, an enlarged left ovary measuring 46 × 51 mm with a well-defined cyst measuring 31 × 36 mm without any septations or fat density was observed (Figure 1). The small bowel, large bowel, ileocaecal junction, uterus, and right adnexa were normal.

A pelvic ultrasound examination performed on Day 5 showed enlarged ovaries with multiple follicles, but there was no evidence of any cyst or free fluid. Therefore, it is thought that there might have been either spontaneous ovulation or regression of the follicle.

Although conservative treatment was applied, the patient presented herself in the outpatient clinic of the surgery department after 16 days due to pain in the right iliac fossa. During physical examination, she was found to have localised tenderness, rebound tenderness, and a positive psoas sign that increased the suspicion of appendicitis. The lab tests showed that the patient’s total leukocyte count was 10,600/mm³, of which neutrophils constituted 55% of the count. Ultrasonography of the right iliac fossa showed that there was a poorly visualised tubular structure, implying that the appendix was inflamed. Therefore, a provisionary diagnosis of appendicitis was made, and the patient was to undergo appendectomy.

**Figure 2:**
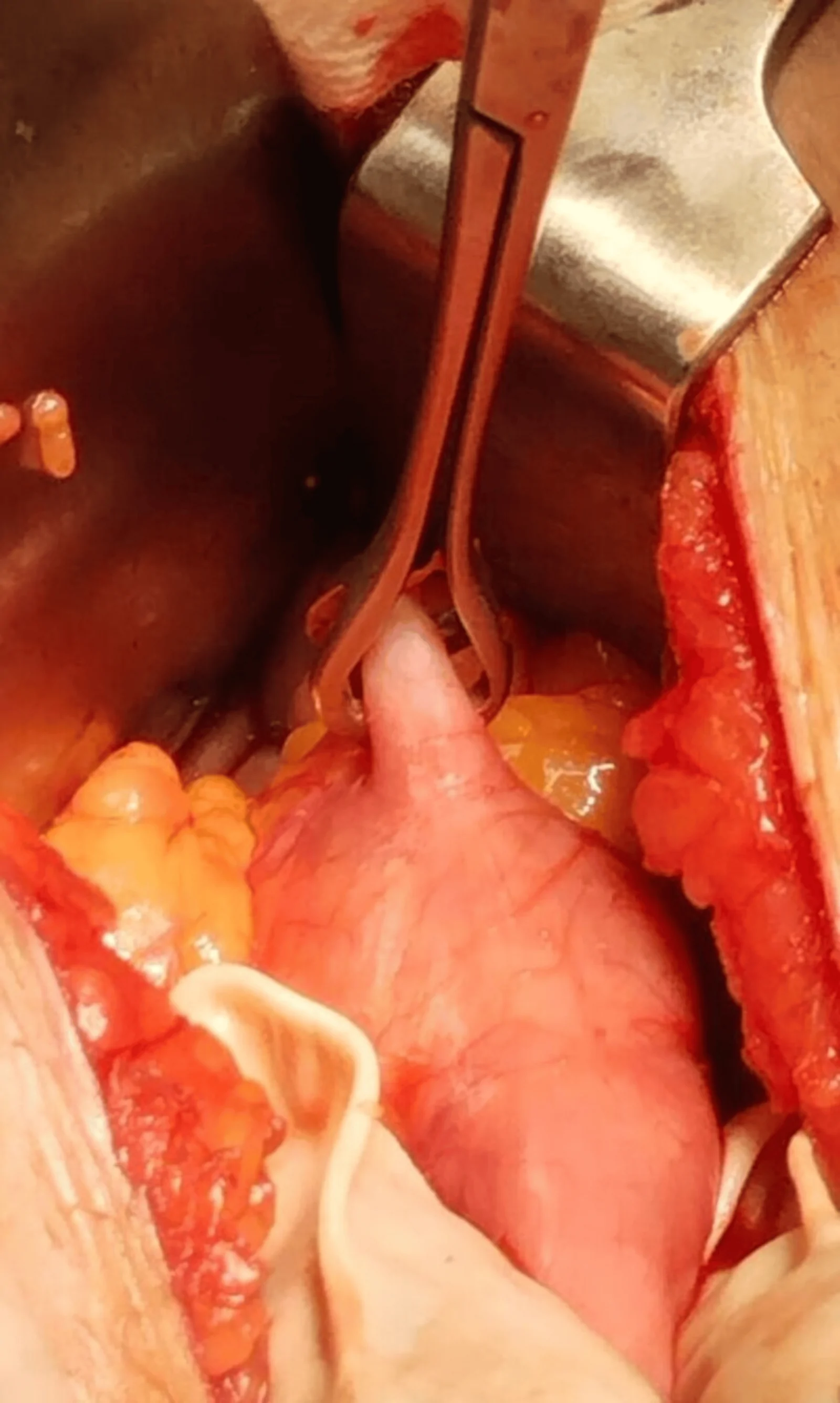
A small nodular structure at the end of the taenia coli, approximately 1 cm in diameter (suspected hypoplastic appendix).

Exploration of the right iliac fossa was done systematically. Taenia coli were traced to their convergence at the base of the caecum, but no appendicectomy could be done since there was no presence of the appendix. There was only a whitish nodule about 1 cm in size (Figure 2). The right colon was mobilised to enable total inspection of the caecum and retrocaecal area. The terminal ileum, ileocaecal junction, pelvis, ovaries, and fallopian tube were examined and found to be normal. There was no finding for the cause of pain in the right iliac fossa, such as Meckel's diverticulum or any other pathology within the abdomen. With no evidence of any pathology amenable to surgery, no further operation was done.

The postoperative course of the patient was smooth. Her abdominal pain subsided conservatively with discharge on oral analgesics. Pelvic ultrasonography revealed no tubo-ovarian pathology. On outpatient follow-up, the wound was found to have healed well. She was haemodynamically stable. While the absence of the vermiform appendix was confirmed intra-operatively, the real cause of her abdominal pain was still unclear with Mittelschmerz and mesenteric lymphadenitis being the two likely diagnoses aside from surgery-related pathology. This case report was prepared in accordance with the CARE (CAse REport) guidelines.

## Discussion

Congenital absence (agenesis) of the vermiform appendix is a relatively infrequent intraoperative finding, with scarce data available in the medical literature. As first documented by Morgagni, it results from the failure in the development of the appendiceal bud in utero or from ischaemia to the appendix, causing regression of the structure [6].

According to Collins, there are five types of appendiceal anomalies, including complete absence, among others. Thus, type I refers to agenesis, whereas the other types include malformation of the caecum along with a rudimentary or absent appendix. It is clinically relevant to differentiate complete agenesis from rudimentary or atrophied appendix, though it may prove difficult intraoperatively without histologic examination [7].

The symptoms of congenital absence of the appendix usually mimic those seen in acute appendicitis, as seen in this case study. They are mainly characterised by right lower quadrant pain, localised tenderness, and signs such as rebound tenderness and the psoas sign [8]. In our patient, the total leukocyte count was 10.7 × 10³/µL, which was at the upper limit of the normal reference range. The combination of symptoms described above often leads to a presumptive diagnosis of appendicitis and surgery for confirmation. Preoperative imaging cannot be used reliably to diagnose agenesis. Both ultrasound and CT scans are intended to detect signs of inflammation, but not the physical presence of the vermiform process [9]. Moreover, in many cases - ours included - preoperative images can show another pathology, e.g., an ovarian mass, adding confusion. Inability to see an intact appendix on imaging scans does not necessarily mean its agenesis.

Literature has also documented similar cases of paediatric patients presenting with symptoms of acute appendicitis, but whose condition was found to be associated with suspected appendiceal agenesis upon surgical exploration. Hammood et al. and Khan et al. also pointed out that preoperative radiological evaluation was not helpful in reaching the diagnosis and that intraoperative examination of the cecum and taenia coli was a must before concluding congenital absence of the appendix [10,11]. This case is comparable to those mentioned above, except for the lack of a histopathological examination that would exclude a rudimentary appendix.

Thus, intraoperative diagnosis becomes the key component in the process. In order to exclude the existence of the appendix, a careful and methodical examination must be carried out by following the course of the taenia coli until their confluence around the base of the cecum. Besides this, the ileocaecal junction should be carefully examined along with the terminal section of the ileum. If no signs of the appendix are found despite careful examination, agenesis may be suspected; still, one must not disregard the possible presence of rudimentary, fibrous, or auto-amputated appendices.

This is due to the presence of a tiny nodule formation at the base of the caecum in this case scenario. Even if there is no definite evidence of appendiceal lumen formation or tubular formation, whether the absence of an appendix can be classified as agenesis or rudimentary appendiceal tissue cannot be conclusively established without histopathological examination; hence, a suspected agenesis or hypoplasia of the vermiform appendix based on peroperative observation was made.

The significance of this uncommon condition, from a practical point of view, is that such a case should not be subjected to extended explorations. Furthermore, this should be recorded in the clinical history of the patient, as the symptoms may recur in the future.

A limitation of this study is the lack of microscopic proof of the nodule at the caecal base, which was discovered intraoperatively. While an exhaustive search did not reveal any presence of the vermiform appendix, leading to the inference that appendiceal agenesis existed, it should still be noted that the presence of a rudimentary or involuted appendix cannot be ruled out entirely.

## Conclusions

The suspected congenital lack of the vermiform appendix and hypoplastic vermiform appendix is an uncommon and often unsuspected intraoperative discovery which may simulate acute appendicitis. The case presented here demonstrates the difficulty in diagnosing right iliac fossa pain when there is ambiguity in the findings on clinical and radiologic studies. A thorough and methodical intraoperative search, which includes following the taenia coli, is required before ruling out the presence of the appendix.

Moreover, this article demonstrates the significance of recognising anatomical anomalies in the differential diagnosis process to prevent unnecessary diagnostic dilemmas. Accurate recording of such cases is necessary for future medical care. While intraoperative findings in the present case support the existence of appendiceal agenesis, it remains possible that some remnant appendicular tissue may exist; thus, proper interpretation of the findings is necessary, and if possible, histopathological evidence should be sought.
